# Sequelae of Septic Arthritis of Hip in a Child Presenting with Acetabular Defect and Hip Dislocation: A Rare Case Report and Literature Review on Successful Treatment with Steel Osteotomy

**DOI:** 10.7759/cureus.42607

**Published:** 2023-07-28

**Authors:** Rajesh Rana, Ashutosh k Nayak, Manmatha Nayak, Abhilash Patra

**Affiliations:** 1 Orthopedics, Institute of Medical Sciences and SUM Hospital, Bhubaneswar, IND; 2 Orthopedics, SCB Medical College & Hospital, Cuttack, IND

**Keywords:** triple innominate pelvic osteotomy, acetabular defect, steel osteotomy, acquired hip dislocation, sequalae of septic arthritis of hip

## Abstract

Septic arthritis of the hip in late childhood leads to different sequelae. These cases are often missed and lead to various disabilities like hip subluxation, limb length discrepancy, and limping. The primary goal is always to reproduce a concentrically reduced stable hip. We are presenting a 13-year child with sequelae of septic arthritis of the hip with dislocation. The child had septic arthritis of the hip two years back. The patient had a superolateral acetabular defect and was treated with triple innominate steel osteotomy. The osteotomy increased the acetabular head coverage and gave a stable congruent hip to the child. Late childhood septic arthritis cases can produce acetabular defects without involving the femoral head leading to dislocation. Such cases can be effectively treated with triple innominate pelvic osteotomy, giving good head coverage with stable congruent hips.

## Introduction

Septic arthritis of the hip in children leads to various sequelae like femoral head epiphyseal damage, subluxation or dislocation of the head, and acetabular damage [1]. All treatments aim to address the pathology and produce a concentrically reduced femoral head in the acetabulum with a stable hip [2]. Choi, Johari, and Hunka's classifications are used to classify the sequelae of septic arthritis of the hip and their management [3-5]. Most classifications are used for sequelae of neonatal sepsis, with the median age of most studies being eight months [6]. There is scanty literature available on pediatric septic arthritis occurring at a later age and its sequelae. Septic arthritis hip leads to the destruction of the head or deformation of the head along with the destruction of joints and acetabulum. These often lead to subluxation or dislocation of the head. In the long term, it may produce arthritic changes in joints. Mitchell et al. showed the results of some sequelae of septic arthritis with acquired hip dislocation [7]. Here we present a case of a child of 13 years with sequelae of septic arthritis. He had septic arthritis two years back.

## Case presentation

**Figure 1:**
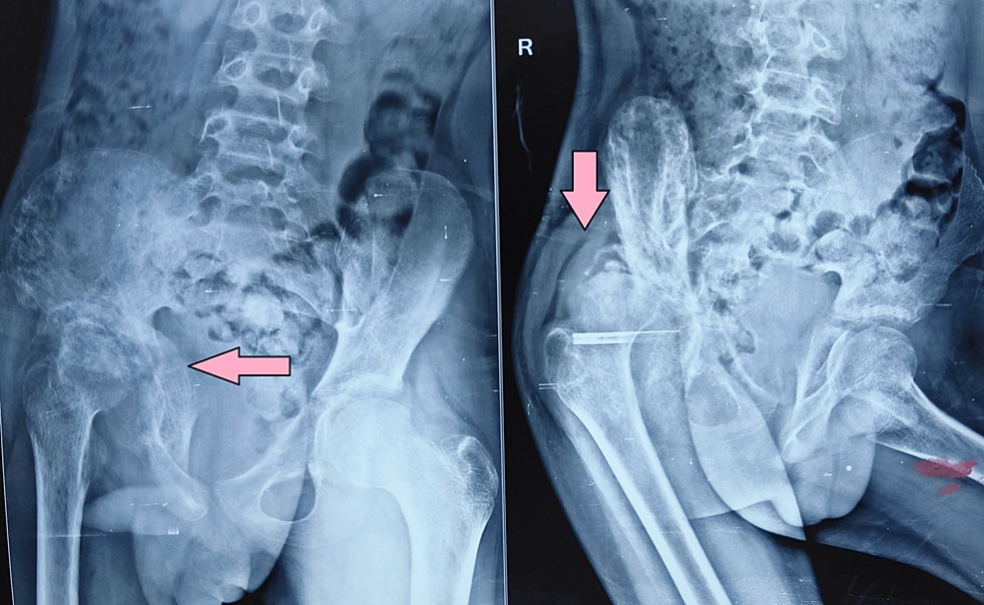
Pre-operative radiograph

A 13-year male child presented to the hospital with a limp and shortened right lower leg. He had a history of septic arthritis right hip two years back, treated by incision drainage and antibiotics. On examination, there was a scar mark over the anterior aspect of the hip. There was no tenderness over the hip joint. Trochanter had gone up on the right side with a normal counter without thickening and broadening. There was muscle wasting around the right thigh and gluteal region. There was a restriction of abduction of the hip with 20 degrees of abduction possible. Adduction of 0 to 30 degrees was possible. Flexion was there from 0 to 100 degrees, and an extension of 10 degrees was possible. Rotations were restricted with an external rotation of 20 degrees possible and an internal rotation of 30 degrees possible. There was a shortening of the right limb of 2 cm was there. Trendelenburg sign was positive. Routine blood investigations to know residual infections were done. Erythrocyte sedimentation rate (ESR), C-reactive protein (CRP), and complete blood count (CBC) were normal, with no signs and symptoms of ongoing infection. In a plain radiograph of the hip, we found the head had gone up and dislocated from the acetabular socket with a bony defect in the acetabulum (Figure 1).

Shenton’s line was broken with increased distance from the teardrop medially. CT scan was done, and it showed a defect in the acetabular superolateral portion leading to head uncoverage and facilitating head dislocation. MRI of the head showed no signs of avascular necrosis yet. The above findings and investigations confirmed it to be sequelae of septic arthritis in a 13-year child with subluxated acetabular head uncoverage because of an infection two years back.

**Figure 2:**
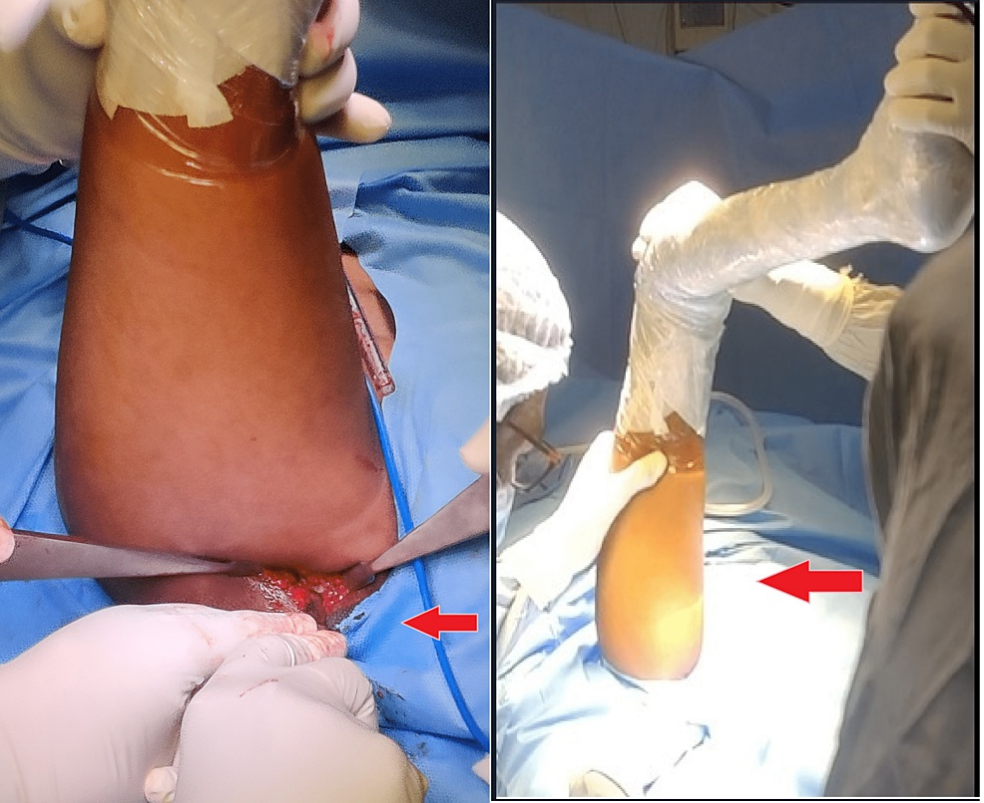
Positioning and approach for ischial ramus osteotomy

The patient was planned for triple innominate osteotomy of steel because of the age of presentation. The patient was given skin traction before surgery. Adductor tenotomy was done to increase the abduction movement. Osteotomy of the ileum was done at the supra-acetabular region, with the cut extending from the anterior inferior iliac spine to the sciatic notch-like salter osteotomy. Similarly, the ischial ramus osteotomy was done at the gluteal region with 90-degree hip flexion (Figure 2).

**Figure 3:**
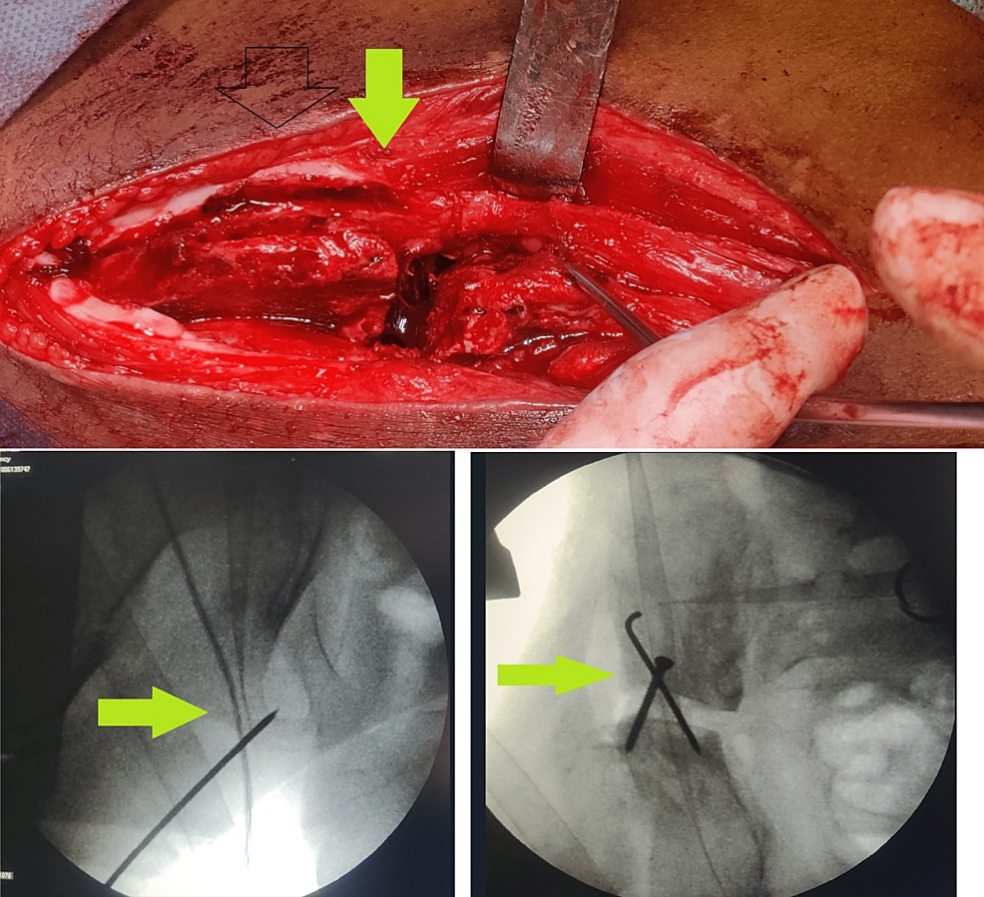
Intra-operative pictures

Osteotomy of the superior ischio pubic rami was done through the same incision of ilium osteotomy. The head of the femur was reduced to its anatomical position in the acetabulum. The acetabular fragment was rotated around the head of the femur to increase superolateral coverage (Figure 3).

**Figure 4:**
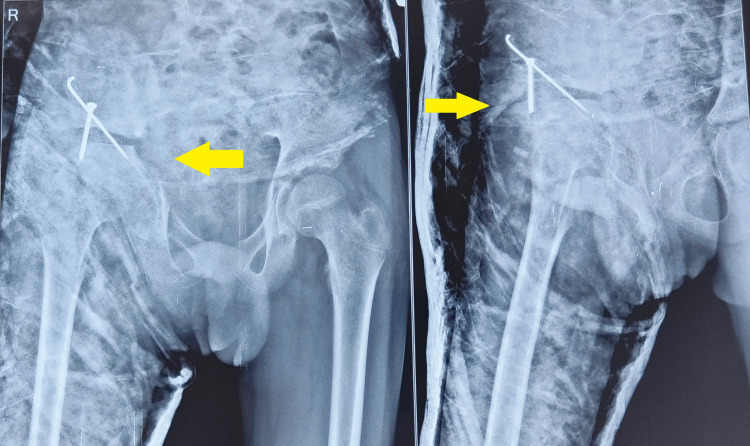
Post-operative radiograph

**Figure 5:**
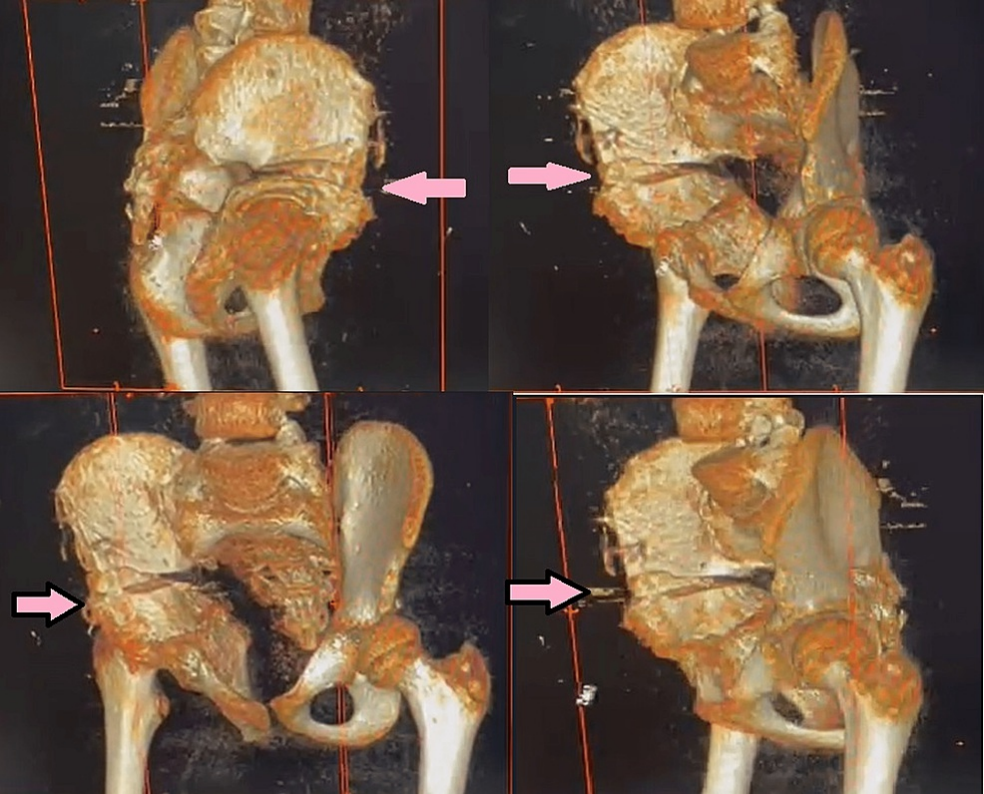
Post-operative CT scan

The acetabular fragment was fixed with a graft from the iliac wing at the ileal osteotomy site. The bone graft was fixed with K wire and one screw (Figure 4). The stability of the hip was checked in all directions, along with congruent reduction. A hip spica cast was applied after the closure of the wound. Post-surgery CT scan was done to know the graft position and acetabular coverage (Figure 5). The hip spica was continued for six weeks, and then partial weight bearing was allowed, followed by full weight bearing. The patient had no further dislocation and had a stable painless hip joint.

## Discussion

Hip involvement by septic arthritis in late childhood has variable presentations. It often leads to damage to the head and acetabulum, leading to version problems of the femoral head and neck and varus or valgus angulation [8]. Fortunately, our case had no femoral head and shaft involvement because of septic arthritis in late childhood. Infantile septic arthritis leads to acetabular dysplasia leading to head uncoverage [9]. In our case, a superolateral acetabular defect led to head subluxation with femoral head uncoverage.

Choi, Johari. and Hunka's classifications are commonly used to stage the post-sequelae of septic arthritis of the hip [10]. These classifications are designed for sequelae of infantile septic arthritis which affects both the femoral head and acetabulum. In our case age of affection of the hip by arthritis was 11 years which led to acetabular defects only. There was no femoral head involvement, and our case does not fit any classifications of the above-mentioned. The adductor tenotomy helped in improving abduction before surgery. Traction before surgery lowered the head and helped in the final operation of containment. In our case, after anesthesia and release, the head was completely reducible congruently in the acetabulum. The reducible and congruent head is the prerequisite for triple innominate osteotomy [11]. The child was in the adolescent age group in our case. Tripple innominate osteotomy allows a high degree of reorientation of the acetabulum and a change of the acetabular version [12]. In our case, we got a good amount of superolateral coverage after reorientating the acetabulum component. Adequate hip abduction movement is a prerequisite for lateral rotation of the acetabulum after osteotomy [13]. Adductor tenotomy helped in increasing the abduction in our case. Many surgeons often do ischial osteotomy in the prone position and turn the patient for iliac osteotomy. In our case, we did the ischial osteotomy in a supine position with flexion of the hip at 90 degrees. We did not find any difficulty in osteotomy, and the advantage was that we did not have to change the draping.

Other types of redirectional osteotomy, like Bernese peri acetabular osteotomy, could have also been done in our case. Bernese osteotomy keeps the posterior column intact, making it a more stable osteotomy [14]. The major drawback of Bernese osteotomy is technical difficulty because of a steep learning curve [15]. Steel osteotomy sometimes produces normal vaginal difficulty, but current literature shows it does not affect much in pregnancy and childbirth [16]. In our case, the patient was a male child.

## Conclusions

In our case, we got satisfactory results in the sequelae of septic arthritis of late childhood cases with steel osteotomy. Septic arthritis of the hip occurring in late childhood can produce different types of defects that only affect the acetabulum. The femoral head may be normal, and such a defect can be managed successfully by steel’s triple innominate osteotomy.
